# Particulate Matter-Induced Emerging Health Effects Associated with Oxidative Stress and Inflammation

**DOI:** 10.3390/antiox13101256

**Published:** 2024-10-17

**Authors:** Eun Yeong Lim, Gun-Dong Kim

**Affiliations:** Division of Food Functionality Research, Korea Food Research Institute (KFRI), Wanju 55365, Republic of Korea; l.eunyeong@kfri.re.kr

**Keywords:** particulate matter, reactive oxygen species, inflammation, oxidative stress

## Abstract

Environmental pollution continues to increase with industrial development and has become a threat to human health. Atmospheric particulate matter (PM) was designated as a Group 1 carcinogen by the International Agency for Research on Cancer in 2013 and is an emerging global environmental risk factor that is a major cause of death related to cardiovascular and respiratory diseases. PM is a complex composed of highly reactive organic matter, chemicals, and metal components, which mainly cause excessive production of reactive oxygen species (ROS) that can lead to DNA and cell damage, endoplasmic reticulum stress, inflammatory responses, atherosclerosis, and airway remodeling, contributing to an increased susceptibility to and the exacerbation of various diseases and infections. PM has various effects on human health depending on the particle size, physical and chemical characteristics, source, and exposure period. PM smaller than 5 μm can penetrate and accumulate in the alveoli and circulatory system, causing harmful effects on the respiratory system, cardiovascular system, skin, and brain. In this review, we describe the relationship and mechanism of ROS-mediated cell damage, oxidative stress, and inflammatory responses caused by PM and the health effects on major organs, as well as comprehensively discuss the harmfulness of PM.

## 1. Introduction

According to studies conducted since the late 1990s, air pollution is associated with respiratory disease, cardiovascular disease (CVD), and lung cancer, with approximately 7 million premature air pollution-related deaths reported each year [1,2]. In the 2019 Global Burden of Disease report, the mortality rate associated with air pollution, a level 2 risk factor, was 11.8%, making it the leading cause of deaths related to environmental pollution at 6.7 million people (95% confidence interval [CI] 5.90 to 7.49) and disability-adjusted life years (DALYs) at 213 million (95% uncertainty intervals [UI] 189 to 240) [3]. Particulate matter (PM), a highly reactive complex of gaseous, liquid, and solid matter, is considered a major cause of air pollution and causes 4.14 million (95% CI 3.45 to 4.80) and 118.2 million (95% UI 95.9 to 138.4) PM-related deaths and DALYs globally in 2019 [4,5]. The health effects of airborne PM exposure have been statistically analyzed in short- or long-term cohorts and meta-analyses of factors such as mortality, hospital admission rates, and changes in lung function [6,7,8,9]. A study of the association between PM and mortality conducted in 20 large cities over 7 years in the United States showed that when PM increased by 10 μg/m^3^, the relative mortality rate increased by 0.51% (95% prediction interval [PI] 0.07 to 0.93) and that the cardiovascular and respiratory system-associated mortality rate increased by 0.68% (95% PI 0.20 to 1.16) [10]. Similarly, a Cancer Prevention II study conducted by the American Cancer Society showed that with each 10 μg/m^3^ increase in PM, the all-cause mortality rate increased to around 4%, and cardiopulmonary- and lung cancer-related deaths increased to 6% and 8%, respectively [11]. Recent cohort studies demonstrated a close association between PM and CVDs, including ischemic heart disease and stroke, as well as respiratory diseases, such as asthma, chronic obstructive pulmonary disease (COPD), lower and upper respiratory tract infections, and cancers of the trachea, bronchi, and lungs [12,13].

**Figure 1:**
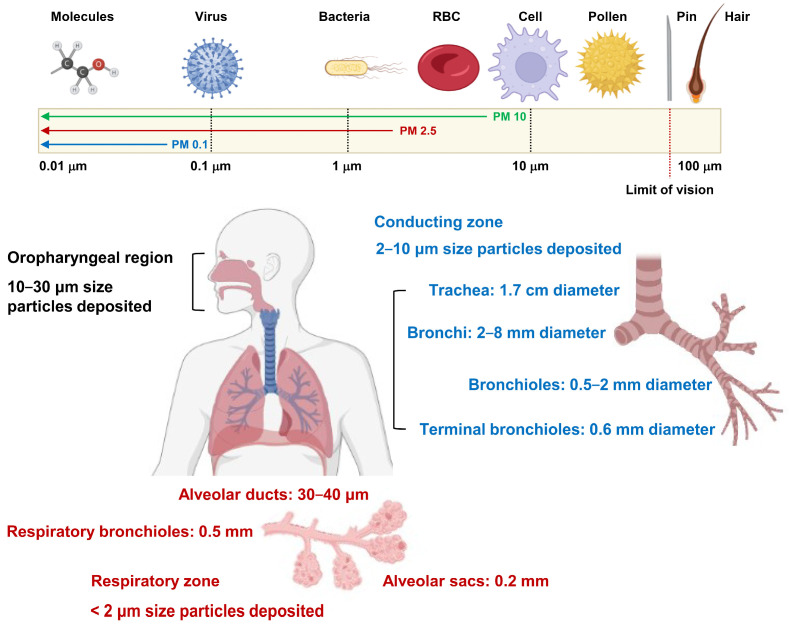
PM classification by aerodynamic particle size and possible routes of penetration and accumulation by particle size in the lungs. Particulate matter: PM; red blood cell: RBC. Created with BioRender.com. accessed on 20 September 2024.

**Figure 2:**
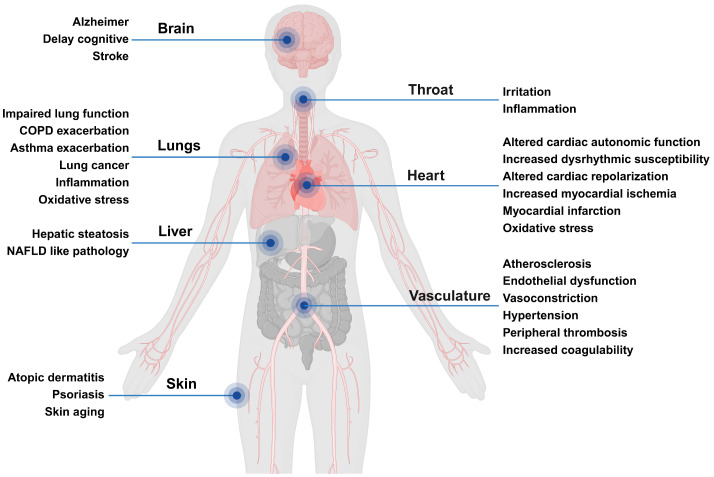
Major organs affected by PM exposure and related diseases and symptoms. Chronic obstructive pulmonary disease: COPD; nonalcoholic fatty liver disease: NAFLD. Created with BioRender.com. accessed on 20 September 2024.

The adverse health effects of PM are primarily attributed to the particle size and constituents. PM10 has an aerodynamic particle size of less than 10 μm, and PM with particle sizes of 5 to 10 μm when inhaled deposit in the trachea and bronchioles (Figure 1) [5]. Particle sizes of 2.5 μm or less and 0.1 μm or less are classified as PM2.5 and ultrafine particles [5]. PM2.5 can penetrate and accumulate in alveolar ducts and capillaries as well as in each organ of the human body through the circulatory system. Depending on the duration of exposure, PM can contribute to the onset and worsening of various diseases (Figure 2) [14,15]. PM can contain aerosol constituents, including carbon monoxide, nitrates, sulfates, ozone, and volatile organic compounds, along with heavy metals, pyrene, and polycyclic aromatic hydrocarbons (PAHs) (Table 1) [16,17]. Interestingly, PM can promote oxidative stress depending on the metal contents, PAHs, particle size, and surface characteristics. Reactive oxygen species (ROS) generated during this process activate intracellular stress signaling molecules such as p38 and c-Jun N-terminal kinases and increase the expression of inflammatory cytokines such as IL1 and IL6 by activating transcription factors, including nuclear factor kappa B (NFκB) and activator protein 1 (AP-1) [18,19]. Recent studies on genome and transcriptome analyses showed that inflammatory responses and oxidative stress induced by exposure to PM are primarily initiated and propagated through epithelial cells and innate immune cells, including neutrophils, monocytes, and macrophages [20,21,22,23]. In particular, the gene ontology category and Kyoto Encyclopedia of Genes and Genomes pathway analyses of macrophages exposed to PM revealed a close association with diverse mechanisms, including inflammatory responses, chemokine signaling pathways, cytokine–cytokine receptor interactions, mitogen-activated protein kinase (MAPK) signaling pathways, and responses to oxygen levels. Accordingly, the expression of genes involved in these signaling pathways such as *Cyp1a1*, *Ptgs2*, *Hmox1*, *Mmp12*, *Nlrp3*, *Cxcr1*, *Cxcl2*, and *Clec7a*, was significantly upregulated [20,21]. PM also causes endoplasmic reticulum stress, apoptosis, ferroptosis, and pyroptosis and can cause DNA damage, epigenetic modifications, and the disruption of homeostasis [24].

This review describes the oxidative stress and inflammatory responses caused by PM and related mechanisms; the health impacts of PM in major areas, including respiratory diseases, CVDs, skin diseases, and neurodegenerative and neurodevelopmental impairments, as well as the mechanisms of these effects are discussed.

## 2. PM-Induced ROS Generation

ROS generation is a major cause of oxidative stress and inflammatory responses according to studies using various disease models related to PM exposure [24]. ROS generation due to PM exposure occurs through the production of hydroxyl radicals mediated by transition metals such as Fe, Ni, and Cu via the Fenton reaction and the hydroxylation of 2′-deoxyguanosine to form 8-hydroxy-2′-deoxyguanosine (8-OHdG) [25]. Furthermore, quinones produced during the metabolism of PAHs by cytochrome P450 (CYP), phagocytic responses mediated by nicotinamide adenine dinucleotide phosphate (NADPH), NADPH oxidases (NOXs) in immune cells activated by PM exposure, and mitochondrial dysfunction contribute to excessive generation of ROS induced by PM exposure [26].

### 2.1. ROS Generation Derived from Metal Components of PM

Valavanidis et al. showed that among PM-constituent metal components, Fe, V, Cu, Cr, Ni, Co, Pb, Cd, Fe, and V increased the production of hydroxyl radicals through the Fenton reaction [27]. In addition, transition metals, including Ni, V, Co, and Cr, generate H_2_O_2_ and highly reactive ROS through the reduction of molecular oxygen in a multivalent state [27]. Fe and Cu, the most abundant metal components of PM, and the transition metals Ni, V, Co, Cr, and Pb induce excessive production of ROS by reducing molecular oxygen mediated by the Fenton reaction, and the augmentation of oxidative damage through 8-OHdG generation increases in proportion to the PM concentration. Similarly, for every 1 μg/m^3^ increase in the concentration of PM containing large amounts of metal components such as V, Mn, Ni, and Pb, the concentration of urinary 8-OHdG in humans significantly increased by 1.67 μg/g (95% CI 0.21 to 3.14) [28]. Interestingly, a recent study showed that following exposure to PM containing Fe (253 μg/g), V (14.7 μg/g), and Ni (76.0 μg/g) co-incubated with the iron chelator deferoxamine, the concentration of 8-OHdG was significantly reduced by approximately 209% compared to that in rat lung epithelial cells exposed to PM only [29]. Collectively, metal ions, including Fe and Cu, in PM cause excessive production of ROS, thereby inducing DNA damage, oxidative stress, and inflammatory responses.

### 2.2. ROS Generation Resulting from Aromatic Compound-Mediated Quinone Production in PM

Quinones are highly reactive, redox-active molecules capable of overproducing ROS, including superoxide, H_2_O_2_, and hydroxyl radicals [30]. They are generated through various mechanisms such as the oxidation of hydroquinones and catechols. These reactions are catalyzed by metal ions and oxidative enzymes such as CYP, peroxidase, cyclooxygenase-2 (COX2), and xanthine oxidase, as well as hydroxylation reactions mediated by CYP, followed by subsequent two-electron oxidation [30]. Benzene, the fundamental structure of aromatic rings, is converted through CYP metabolism into catechols or hydroquinones, which are ultimately oxidized to o- and p-quinones [31,32]. Polychlorinated biphenyls are also converted into o- and p-quinones via catalyzed aromatic hydroxylation reactions [33]. Compared with p-quinones, o-quinones are short-lived, highly reactive, and exhibit various biological activities [34]. A recent study showed that o-quinones contribute to neurodegeneration, neurotoxicity, and dermal toxicity by increasing oxidative stress and inducing dysfunction in proteins and mitochondria [30]. PAHs are metabolized to o-quinones via a complex mechanism involving the initial formation of arene oxides mediated by CYP, the ring opening catalyzed by epoxide hydrolase, catechol formation mediated by aldo-keto reductase, and two-electron oxidation [26,35]. Additionally, aldo-keto reductase catalyzes the NADPH-dependent reduction of o-quinones, which facilitates excessive production of ROS. PAHs and their various derivatives also induce macrophage activation, oxidative stress, and DNA damage mediated by 8-OHdG [35]. The major constituents of PM, such as PAHs and their oxidative derivatives, including 9,10-anthraquinone and 7,12-benz(a)anthraquinone, significantly increased ROS generation and migration in vascular smooth muscle cells; these effects were significantly inhibited by the antioxidant N-acetylcysteine [36]. Sun et al. showed that PAHs such as pyrene, anthracene, and benzo(a)anthracene derived from solid fuel combustion and containing three-ring and four-ring structures are closely associated with the increased generation of ROS, cellular toxicity, and inflammatory cytokine production [37]. Furthermore, a recent study identified that exposure to 16 PAHs designated as priority by the United States Environmental Protection Agency, including naphthalene, fluorene, pyrene, chrysene, and benzo(a)pyrene, induced ROS and 8-OHdG generation, cytotoxicity, and morphological alterations in human umbilical vein endothelial cells [38].

### 2.3. Oxidase-Mediated ROS Generation Induced by PM Exposure

Exposure to PM induces the generation of ROS, inducible nitric oxide synthase, and inflammatory cytokines in monocytes and macrophages via innate immune responses [39]. Additionally, phagocytic activity in macrophages enhances the production of superoxide anions mediated by NOXs, which contributes to the generation of reactive nitrogen species (RNS) such as peroxynitrite [40]. Phagocytosis is mainly mediated by the activation of NOX2, which consists of four cytosolic components, namely neutrophil cytosolic factor (NCF) 1, NCF2, NCF4, Rac family small GTPase 2, and transmembrane proteins including p22phox and gp91phox [41,42]. Upon the initiation of phagocytosis, the cytosolic subunit Rac2 is activated by Rac guanine nucleotide exchange factors, enabling GTP binding [43]. Subsequently, the transmembrane heterodimer p22phox and gp91phox translocate to the phagosomal membrane, where they associate with a trimeric complex composed of NCF1, NCF2, and NCF4 [44]. The phosphorylation of NCF1 and NCF2 facilitates structural changes and stabilization mediated by GTP-Rac2, p22phox, and gp91phox, leading to the overproduction of myeloperoxidase and superoxide anion radicals (O_2_^−^) [45]. This, in turn, contributes to the generation of ROS such as hydroxyl radicals, H_2_O_2_, and hypochlorous acid [45]. Similarly, PM exposure upregulates NOX1 and NOX2, resulting in excessive infiltration of inflammatory cells, including macrophages, and increased oxidative stress in an ischemia–reperfusion lung injury model [46]. Jin et al. identified that diphenyleneiodonium, a NOX inhibitor, suppresses ROS generation, mitochondrial damage, and apoptotic cell death induced by PM exposure in human bronchial epithelial cell lines and rats [47]. Furthermore, PM exposure increases the expression of dual oxidase 1, which is mediated by the overexpression of NOX1 and NOX4 in human keratinocytes, thereby promoting Ca^2+^ channel activation in the cytoplasmic membrane or endoplasmic reticulum and resulting in increased ROS generation [48]. Kampfrath et al. showed that PM exposure significantly increased Toll-like receptor (TLR) 4 and NOX activation-mediated ROS generation as well as the production of oxidized phospholipid derivatives of 1-palmitoyl-2-arachidonyl-*sn*-glycero-3-phosphorylcholine in Ly6C monocytes in a mouse model of systemic vascular dysfunction [49].

### 2.4. Mitochondrial Dysfunction-Mediated ROS Generation Induced by PM Exposure

The disruption of mitochondrial homeostasis and dysfunction primarily arise from the incomplete reduction of molecular oxygen (O_2_) to H_2_O in the electron transport chain due to electron leakage, leading to the generation of ROS [50,51]. Mitochondrial dysfunction associated with PM exposure includes mitochondrial swelling, cristae damage, mitochondrial DNA damage, impaired mitophagy, and excessive ROS production [52]. Naav et al. showed that PM entered trimester trophoblast cells, causing mitochondrial vacuolization, damage to cristae morphology, and increased H_2_O_2_ production and protease activity [53]. Similarly, in a recent study, PM exposure induced dysfunction in 16HBE human airway epithelial cells by increasing mitochondrial ROS and decreasing mitochondrial oxygen consumption and the mitochondrial membrane potential [54]. Yang et al. showed that PM exposure causes dysfunction in human lung epithelial BEAS-2B cells by reducing the mitochondrial membrane potential and DNA copy number and increasing ROS generation, damage to DNA strands, and expression of the inflammatory cytokine IL1β [55]. Mitophagy is a homeostasis maintenance strategy that prevents mitochondrial damage caused by exposure to allergens, stress, and pollutants, such as PM [56]. The molecular mechanisms underlying mitophagy are categorized into two main pathways. The receptor-mediated pathway involves various outer mitochondrial membrane proteins such as BCL2-interacting protein 3, Fun14 domain-containing protein 1, and Nip3-like protein X, which directly interact with LC3 [57]. The PTEN-induced kinase 1 (PINK1)-Parkin-mediated pathway involves the recruitment of polyubiquitinated outer mitochondrial membrane proteins and adaptor molecules, such as p62 and nuclear domain protein 52, which then bind to LC3 to promote the formation of autophagosomes [58]. Qiu et al. showed that PM exposure induced ROS and RNS generation, mitochondrial fission, and PINK1-Parkin-mediated mitophagy while inhibiting mitochondrial transmembrane potential in primary stellate cells and cell lines [59]. Furthermore, a recent study showed that PM exposure leads to abnormal structural changes, including mitochondrial swelling and cristae disorder in alveolar macrophages, as well as excessive ROS production mediated by respiratory bursts and mRNA expression of mitochondrial fission and fusion-associated genes, including *Opa1*, *Mfn1*, *Mfn2*, *Fis1*, and *Drp1* in Sprague Dawley rats [60].

## 3. PM-Associated Respiratory Disease

**Figure 3:**
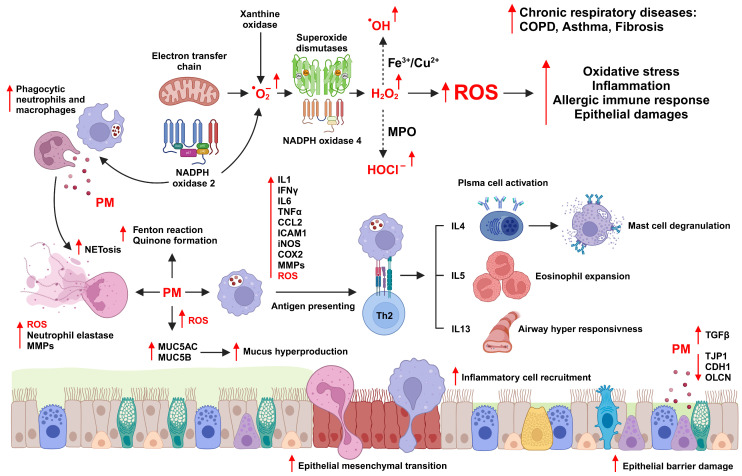
Onset and exacerbation pathogenesis of chronic inflammatory respiratory disease by PM exposure and associated ROS signaling pathway. C-C motif chemokine ligand 2: CCL2; cadherin 1: CDH1; chronic obstructive pulmonary disease: COPD; cyclooxygenase 2: COX2; intercellular adhesion molecule 1: ICAM1; interferon gamma: IFNγ; interleukin: IL; inducible nitric oxide synthase: iNOS; matrix metalloproteinase: MMP; myeloperoxidase: MPO; mucin 5AC: MUC5AC; mucin 5B: MUC5B; nicotinamide adenine dinucleotide phosphate: NADPH; occludin: OLCN; particulate matter: PM; reactive oxygen species: ROS; transforming growth factor beta: TGFβ; tight junction protein 1: TJP1; tumor necrosis factor alpha: TNFα. Created with BioRender.com. accessed on 20 September 2024.

Ambient PM can directly penetrate the body through the respiratory tract; thus, most research on the health effects of PM has focused on respiratory diseases [61,62]. Short-term exposure to PM causes shortness of breath, chest pain, and coughing, whereas long-term exposure can inhibit lung development in young children and chronically inhibit lung growth and function [63,64]. Several studies showed that with a 150 μg/m^3^ increase in PM exposure, lung function decreased by 3% to 6%, and that the concentration of PM in the surrounding environment is highly correlated with the induction of asthma and COPD [65,66]. PM induces airway epithelial damage via oxidative stress [67]. Intracellular ROS trigger the release of inflammatory cytokines through the NOD-like receptor family pyrin domain-containing 3 (NLRP3) inflammasome, AP-1, and MAPK signaling pathways, leading to lung damage and the exacerbation of respiratory diseases [52] (Figure 3).

### 3.1. PM-Induced Oxidative Stress in Asthma

According to the 2019 Global Burden of Disease report, the global prevalence of asthma is 262.4 million (95% UI 224.05 to 309.45), the number of deaths is 461.1 thousand (95% UI 366.58 to 559.01), and the number of DALYs is 21.5 million (95% UI 17.14 to 26.97) [68]. Asthma is a complex allergic disease characterized by chronic airway inflammation and oxidative stress; eosinophils, lymphocytes, neutrophils, and mast cells produce inflammatory mediators, ROS, and RNS, which negatively affect the redox balance [69]. The focus on the role of ROS in asthma stems from the fact that environmental pollutants such as ozone, cigarette smoke, and PM are associated with increased asthma incidence and severity. Most of these pollutants possess oxidative properties, and inflammatory cells associated with the pathophysiology of asthma also generate ROS [69,70]. Oxidative stress plays an important role in asthma development. H_2_O_2_ was detected at higher levels in the exhaled breath of patients with asthma than in healthy individuals, and the severity of asthma increased with increasing concentrations of H_2_O_2_ [71]. More superoxide anions are produced in macrophages isolated from the airways of patients with asthma, and ROS production increases in eosinophils upon antigen stimulation [72,73]. Many antioxidants in cells, such as superoxide dismutase, glutathione peroxidase, catalase, and peroxiredoxin, process the generated oxygen radicals [69]. Among these antioxidants, peroxiredoxin reacts the fastest with oxygen radicals, which are transformed into an irreversible hyperoxidized form [74]. In the peripheral blood mononuclear cells of patients with asthma, hyperoxidized peroxiredoxin and peroxiredoxin ratios were higher than those in normal individuals, and more oxygen radicals were generated after H_2_O_2_ treatment [75]. These results suggest that peroxiredoxin activity to handle free radicals in patients with asthma is reduced compared with that in normal individuals, exposing them to oxidative stress.

Many studies showed that PM exposure causes an increased inflammatory response in airway epithelial cells, increases airway hyperresponsiveness, worsens the symptoms of asthma and COPD, and increases emergency department visits [76,77]. A time-stratified case-crossover study linking Danish national registry data with air pollution measurements demonstrated that among registered children and adolescents aged 0–18 years, 8226 hospital admissions were related to asthma. For each one-unit increase in the interquartile range of the 5-day average of PM10, the odds ratio (OR) for asthma-related hospital admissions was 1.07 (95% CI 1.03 to 1.12) [78]. Fan et al. found that for every 10 μg/m^3^ increase in the PM concentration, the incidence of asthma increased by 1.05% (95% CI 1.00 to 1.11), and a stratified analysis revealed a significant increase in asthma prevalence among infants under 4 years old, with an OR of 2.02 (95% CI 1.35 to 3.03) and among the elderly population aged 75 and above, with an OR of 1.39 (95% CI 1.09 to 1.79) [79]. Additionally, a meta-analysis of 41 studies showed that the risk of developing asthma in children per 1 μg/m^3^ increase in PM2.5 exposure was 1.03-fold (95% CI 1.01 to 1.05) and per 2 μg/m^3^ increase in PM10 exposure (95% CI 1.02 to 1.08) was 1.05-fold [80]. Another meta-analysis exhibited that a short-term increase of 10 μg/m^3^ in PM2.5 concentration is associated with a 1.10% increase in respiratory disease-related mortality (95% CI 0.59 to 1.62%) [81].

PM exposure contributes to oxidative stress, inflammation, and immune responses in asthma. In a cross-sectional analysis, the risk of asthma increased with traffic intensity (OR 1.09, 95% CI 1.00 to 1.18 per 5000 vehicles/day), and the increase in PM2.5 exposure mediated by traffic intensity was associated with elevated exhaled concentrations of 8-isoprostane, a marker of oxidative stress [82]. Additionally, co-stimulation with PM2.5 and the house dust mite allergen Der p1 increased the production of malondialdehyde (MDA) and mRNA and protein expression of IL25, IL33, and CCL17, which are hallmarks of innate immunity and asthma, in a human bronchial epithelial cell line. Similarly, PM exposure was recently found to increase inflammatory cell infiltration in bronchoalveolar lavage fluid and inflammatory responses in lung tissue, including the bronchiolar, perivascular, and alveolar regions, and upregulated the plasma levels of asthma-associated markers such as IgE, IL5, and IL25 in an asthma mouse model established by co-stimulation with PM and ovalbumin (OVA) [83]. Through abiotic and biotic assays using offline fractional exhaled nitric oxide and macrophage ROS, which are biomarkers of airway inflammation, Delfino et al. found that the oxidation potential following PM exposure was related to airway inflammation in pediatric patients with persistent asthma [84]. Furthermore, oxidative stress induced by PM exposure damages the airway epithelium and contributes to the exacerbation of asthma. A recent study showed that exposing human bronchial epithelial cells to PM increased ROS-dependent NFκB-mediated fibronectin production; transforming growth factor β (TGFβ) expression; and mRNA expression of epithelial-to-mesenchymal transition markers such as ACTA2, SNAIL1, and SNAIL2 [85]. Wang et al. found that PM exposure caused p38 MAPK- and heat shock protein 27-mediated endothelial cell barrier disruption by increasing oxidative stress in endothelial cells derived from the human pulmonary artery, which is ultimately associated with lung dysfunction [86]. Additionally, PM2.5 exposure increased the activation of NFκB and MAPK signaling pathway-mediated ROS production, the inflammatory response, and airway epithelial barrier-associated gene expression such as tight junction protein 1 and E-cadherin in BEAS-2B cells [87]. Additionally, in an asthma mouse model established through co-stimulation with PM and OVA, PM exposure caused airway barrier dysfunction, resulting in increased leakage of serum proteins from the blood vessels into the airways [87]. Through transcriptome analysis and a gene set enrichment analysis of RNA sequencing data in an OVA-induced asthma mouse model exposed to PM2.5, Zhang et al. showed that stanniocalcin 2 plays a key role in regulating the epithelial barrier in response to PM exposure [88]. Accordingly, this study showed that ROS production, which was upregulated by exposure to PM2.5, decreased trans-epithelial electrical resistance and mRNA expression of *Tjp1*, *Cdh1*, and *Ocln* and led to overexpression of the inflammatory response and mucus secretion in the airway of mice [88].

Additionally, oxidative stress induced by PM exposure leads to mucus hyperproduction, which is a major characteristic of asthma. In a recent study, PM2.5 exposure increased the production of MDA, myeloperoxidase, and ROS, along with an increased number of goblet cells in the bronchial epithelium as well as upregulated mucin 5 subtype AC via the Sestrin2/Kelch-like ECH-associated protein 1 (Keap1)/nuclear factor erythroid 2-related factor 2 (Nrf2) pathway in an OVA-induced mouse asthma model [89]. Additionally, He et al. showed that neutrophil extracellular traps, neutrophil infiltration, mucus hypersecretion mediated by PM2.5, and exposure-induced ROS were closely associated with the upregulation of NADPH quinone dehydrogenase 1 based on transcriptome analysis of mild-to-severe asthma datasets [90].

### 3.2. PM-Induced Oxidative Stress in COPD

COPD is a chronic progressive disorder characterized by an irreversible airflow limitation, primarily caused by repeated exposure to harmful particles such as cigarette smoke, toxic gasses, and PM [91]. According to the 2019 Global Burden of Disease report, the global mortality rate for COPD was 35.4%, with a prevalence of 212.3 million (95% UI 200.4 to 225.1); COPD was associated with 3.3 million deaths (95% UI 2.9 to 3.5) and 74.4 million DALYs (95% UI 68.2 to 80.1) [92]. In 2019, PM was the leading risk factor for COPD [93]. The number of deaths attributable to PM-associated COPD was 695.1 thousand (95% UI 553.83 to 861.86), with 15.4 million (95% UI 12.39 to 18.96) associated DALYs [93]. A cross-sectional analysis of complete covariate data and lung function measurements from the UK Biobank revealed that for each 5 μg/m^3^ increase in PM2.5 concentration, the forced expiratory volume in 1 s (FEV_1_), forced vital capacity (FVC), and FEV_1_/FVC ratio decreased by −83.1 mL (95% CI −92.50 to −73.75), −62.6 mL (95% CI −73.91 to −51.32), and −2.27 (95% CI −2.57 to −1.96), respectively [94]. Accordingly, the prevalence of COPD increased by 1.52% (95% CI 1.42 to 1.62) with PM2.5 exposure and by 1.08% (95% CI 1.00 to 1.16) with PM10 exposure [94]. These findings demonstrate an association between PM exposure, decreased lung function, and COPD prevalence. Similarly, a large-scale longitudinal cohort analysis of long-term exposure to PM2.5 revealed that each 5 μg/m^3^ increase in PM2.5 concentration was associated with a decrease in FEV_1_ by 1.46% (95% CI 1.47 to 1.60), FVC by 1.18% (95% CI 1.14 to 1.21), and the FEV_1_/FVC ratio by 0.21% (95% CI 0.19 to 0.22) [95].

Harmful substances such as PM and allergens are inhaled into the lungs; they generate ROS or other oxygen radicals that induce oxidative stress, which are primarily produced by inflammatory and epithelial cells that constitute the airways [96]. The key enzymes responsible for ROS production are NOXs. In patients with COPD, an imbalance between oxidants and antioxidants caused by NOXs leads to oxidative stress and inflammation within cells [97]. This inflammatory response damages the defense mechanisms of the airways and blood vessels, attracting additional inflammatory cells to the lungs and further exacerbating the oxidative–reduction imbalance, which in turn induces NFκB-dependent inflammation [98]. Chronic inflammation and oxidative stress result in the destruction of alveolar cells, which is exacerbated by growth factor deficiency, oxidative stress, and apoptosis [52]. This destruction contributes to emphysema and fibrosis, leading to airway remodeling and obstruction [98].

PM-induced oxidative stress plays a crucial role in the pathophysiology of COPD by contributing to inflammation, the immune response, emphysema, and mucociliary clearance [91]. Chandel et al. showed that intratracheal PM2.5 exposure promoted inflammatory cytokines and chemokine production such as CXCL1, IL6, TNFα, IL1β, G-CSF, and CCL2 in bronchoalveolar lavage fluid, as well as elevated oxidative stress in lung tissues, increased mitochondrial ROS in inflammatory cells, and enhanced emphysematous lesions in mice [99]. Similarly, a recent study demonstrated that PM exposure led to the development of airway fibrosis characterized by increased α-smooth muscle actin, larger total wall areas of the small airways, and higher hydroxyproline contents in the lung tissue, as well as increases in goblet cell hyperplasia, mucus hyperproduction, excessive inflammatory cell recruitment, and oxidative stress biomarker levels such as MDA and 8-OHdG in rats [100]. Wang et al. showed that PM exposure increases the production of oxidative stress- and inflammation-associated markers such as ROS, mitochondrial ROS, MDA, and COX2 in patients with COPD and in in vitro and in vivo analyses, causes emphysema and airway inflammation by upregulating the expression of the ferroptosis-related markers LC3B, NCOA4, and FTH1 [101]. Furthermore, Liu et al. observed elevated levels of plasma IL6 and ICAM1 in patients with COPD. Consistent with these observations, PM-exposed lung epithelial cells and mice exhibited increased ROS production, along with upregulated ICAM1 mediated through the IL6/AKT/STAT3/NFκB signaling pathway [102].

### 3.3. PM-Induced Oxidative Stress in Pulmonary Fibrosis

Pulmonary fibrosis is characterized by abnormal degradation or remodeling of the extracellular matrix, leading to excessive accumulation of collagen, fibrin, and other matrix components, resulting in irreversible damage to the distal lungs. This pathological process impairs normal gas exchange, causing pulmonary dysfunction and failure [103,104]. Oxidative stress induced by a redox imbalance of ROS and RNS leads to cellular dysfunction and damage to the pulmonary epithelial tissue, thereby promoting the development of fibrosis [105]. In addition, growth factors and cytokines produced by pulmonary inflammatory cells, including macrophages and neutrophils, generate high levels of ROS and RNS, which further contribute to fibrosis [105]. A recent study showed that short-term exposure to PM2.5 promoted collagen deposition, the uptake of fluorodeoxyglucose F18, contents of hydroxyproline, MDA, E-cadherin, and fibronectin, which are associated with the onset and progression of pulmonary fibrosis, and induced mRNA expression including *Tnf*, *Il1β*, *Il6*, *Ccl2*, and *Icam1*, resulting in a chronic oxidative stress-mediated epithelial–mesenchymal transition in rats [106]. Furthermore, PM2.5 exposure promotes TGFβ activation in alveolar type II cells, leading to increased cortical cell stiffness, cell elongation, reduced circularity, and elevated intracellular ROS levels [107]. These results suggest that extracellular matrix stiffness, mediated by activation of TGFβ, is associated with excessive production of ROS due to PM exposure. Han et al. revealed that an epitranscriptomic analysis of a PM2.5-induced pulmonary fibrosis mouse model was associated with increased ROS production and aberrant mRNA 5-methylcytosine levels in fibrotic lung tissue [108]. Accordingly, genes related to oxidative stress, the inflammatory response, and immune response such as *Adipoq*, *Atp5l*, *Atpif1*, *Atp5j2*, *Chi3l1*, *Fgr*, *Lcn2*, *Mmp9*, *Ndufb6*, *Slc11a1*, and *Tyrobp* are 5-methylcytosine gain-upregulated factors that contribute to the development of pulmonary fibrosis [108].

Idiopathic pulmonary fibrosis is a chronic progressive fibrotic interstitial pneumonia of unknown etiology more commonly observed in males. South Korea has the highest global prevalence of idiopathic pulmonary fibrosis, with an adjusted prevalence estimate of 0.57 to 4.51 per 10,000 individuals (95% CI 2.99 to 6.79) [109,110]. In North America, the prevalence ranges from 2.40 to 2.98 per 10,000 individuals (95% CI 1.33 to 5.19), whereas in Europe, the prevalence is estimated to be between 0.33 and 2.51 per 10,000 individuals (95% CI 0.21 to 4.05) [109]. A recent study demonstrated that treatment with the antioxidant N-acetylcysteine inhibited PM-induced ROS production in human bronchial epithelial cells and suppressed ROS-dependent fibronectin and TGFβ production [85]. These findings indicate that ROS inhibition has therapeutic potential for the treatment of PM-induced idiopathic pulmonary fibrosis.

## 4. PM-Associated CVD

The effects of PM2.5 on CVDs have been revealed through various clinical and preclinical studies. Epidemiological and cohort studies demonstrated a correlation between exposure to ultrafine and fine PM and various CVD phenotypes, including hypertension, ischemic heart disease (IHD), heart failure, myocardial infarction (MI), and stroke [111,112,113,114,115,116]. Various meta-analyses demonstrated that PM exposure increases the incidence of CVD. For instance, according to a meta-analysis of 84 cohort studies involving 28,215,394 participants, an increase in PM2.5 exposure was associated with elevated risks of CVD (hazard ratio [HR] 1.10, 95% CI 1.02 to 1.19), stroke (HR 1.13, 95% CI 1.06 to 1.19), hypertension (HR 1.07, 95% CI 1.01 to 1.14), all-cause mortality (HR 1.06, 95% CI 1.04 to 1.09), CVD mortality (HR 1.10, 95% CI 1.07 to 1.12), and ischemic heart disease (IHD) mortality (HR 1.11, 95% CI 1.07 to 1.16). Increased PM10 exposure was associated with heart failure (HR 1.25, 95% CI 1.04 to 1.50), all-cause mortality (HR 1.16, 95% CI 1.06 to 1.27), CVD mortality (HR 1.17, 95% CI 1.04 to 1.30), and IHD mortality (HR 1.03, 95% CI 1.01 to 1.05) [111]. A 10 μg/m^3^ increase in PM2.5 concentration was associated with cardiovascular health, specifically with composite CVD (HR 1.06, 95% CI 1.00 to 1.12), acute coronary events (HR 1.14, 95% CI 1.12 to 1.17), stroke (HR 1.11, 95% CI 1.06 to 1.17), hypertension (HR 1.07, 95% CI 1.03 to 1.11), all-cause mortality (HR 1.05, 95% CI 1.03 to 1.07), cardiovascular mortality (HR 1.09, 95% CI 1.05 to 1.13), and IHD mortality (HR 1.15, 95% CI 1.10 to 1.20). Additionally, another meta-analysis of 42 studies revealed that for every 10 µg/m^3^ increase in long-term PM2.5 exposure, IHD mortality increased by 23% (95% CI 15% to 31%), cerebrovascular mortality by 24% (95% CI 13% to 36%), incident stroke by 13% (95% CI 11% to 15%), and MI by 8% (95% CI 1% to 18%), although the increase in MI was not significant [112]. Both studies investigated IHD mortality; however, the differences in their reported values may be attributed to variations in exposure duration. Several cohort studies confirmed the relationship between PM exposure and CVD. A study conducted in 1993 confirmed that exposure to PM2.5 and hospitalization/death due to CVD had a linear relationship [1]. Cohort studies in the United States and China revealed that long-term exposure to PM2.5 increases the risk of CVD. For each 10 µg/m^3^ increase in PM2.5 exposure, the HR for CVD incidence was 1.251 (95% CI 1.22 to 1.28) and for mortality was 1.164 (95% CI 1.11 to 1.21) [113]. Notably, another long-term study of exposure to PM2.5 in 65,893 postmenopausal women residing in metropolitan areas in the United States revealed a 24% increase in cardiovascular events for a 10 µg/m^3^ increment of PM2.5. Further analysis revealed that the incidence of coronary heart disease increased by 21%, cerebrovascular disease by 35%, MI by 6%, coronary revascularization by 20%, and stroke by 28% [114]. Additionally, exposure to PM2.5 was associated with a 76% increase in CVD-related mortality. A recent study predicted that with PM2.5 levels reduced to 4 µg/m^3^, cardiovascular-related deaths could be reduced by 12.4% [115]. Even though the average annual exposure to PM2.5 was below 12 µg/m^3^, it was observed that for every 10 µg/m^3^ increase in PM, the HR for cardiovascular mortality, acute MI, and stroke was 2.31, 1.51, and 1.41, respectively [116].

### 4.1. Mechanisms of CVD Induced by PM

**Figure 4:**
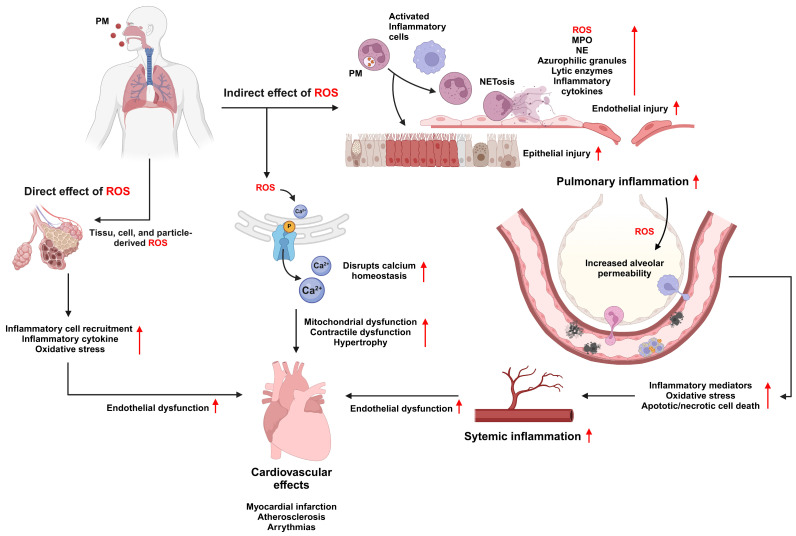
Effects and major pathways of ROS-mediated pathophysiology on cardiovascular diseases following PM exposure. Myeloperoxidase: MPO; neutrophil elastase: NE; particulate matter: PM; reactive oxygen species: ROS. Created with BioRender.com. accessed on 20 September 2024.

PM affects the cardiovascular system through three interconnected pathways. The direct entry of PM into the bloodstream and systemic oxidative stress indirectly trigger inflammation and disrupt the autonomic nervous system. PM can cross the pulmonary epithelium and enter the bloodstream to generate ROS, elicit inflammatory responses, and disrupt calcium homeostasis, thereby exerting profound effects on cardiovascular function. Inhaled PM can also penetrate deep into the respiratory tract, reaching the alveoli in the lungs and releasing inflammatory mediators. These inflammatory mediators not only promote local inflammation in the lungs but also enter the bloodstream, initiating a systemic inflammatory response. Finally, PM exposure affects the autonomic nervous system, impairing the heart rate and increasing the risk of cardiovascular dysfunction. In all these pathways, ROS production plays a crucial role in mediating cardiovascular damage (Figure 4).

Although ROS are essential mediators of cellular signaling and defense mechanisms, excessive ROS production leads to oxidative stress, causing high levels of cellular damage. Oxidative stress compromises the integrity of cellular membranes, proteins, and DNA, leading to necrosis and apoptotic cell death, which can result in serious pathological conditions including heart failure [117]. Excessive ROS production significantly impairs the availability of NO, a critical molecule involved in vasodilation and vascular homeostasis maintenance. Given the critical function of NO in vascular dilation and the maintenance of proper blood flow, a decrease in NO can lead to vasoconstriction, contributing to elevated blood pressure and arterial hypertension development. For instance, elevated levels of ROS, oxidative stress markers, MDA, and inflammatory markers such as TGFβ and IL6 have been detected in both the lung and heart tissues following PM exposure [118]. Oxidative stress in the heart compromises cardiomyocyte function, damages mitochondria, reduces ATP production, and impairs contractility. These effects further predispose the heart to conditions such as hypertrophy, arrhythmia, and heart failure. In addition, oxidative stress triggered by PM in the lungs and propagated systemically can activate TLR4/Myd88/NFκB, which are critical transcription factors in the regulation of genes related to inflammation such as NLRP3 inflammasome [119]. These changes can result in the persistent production of inflammatory mediators, creating a vicious cycle of oxidative stress and inflammation that affects multiple organs.

A key protective mechanism against oxidative stress is the activation of Nrf2, a redox-sensitive transcription factor that promotes the expression of antioxidant and cytoprotective genes. Nrf2 activation has the potential to prevent or treat CVDs, such as ischemia/reperfusion injury and diabetic cardiomyopathy [120,121]. Under normal conditions, Nrf2 binds to Keap1 in the cytoplasm [122]. Upon stimulation, Nrf2 dissociates from Keap1, translocates to the nucleus, and promotes the expression of cytoprotective genes including heme oxygenase 1, NADPH quinone dehydrogenase 1, and glutamate-cysteine ligase modifiers [123]. Moreover, Nrf2 induces the expression of anti-inflammatory and anti-fibrotic genes, neutralizes free radicals, and eliminates toxins [124]. However, in PM2.5-induced diseases, Nrf2 signaling is downregulated, leading to increased inflammatory responses [125,126]. Another significant mechanism by which PM induces cardiovascular dysfunction is calcium imbalance. The generation of ROS and calcium is linked to cardiovascular dysfunction induced by PM [127,128]. Ca^2+^ is essential for regulating cardiac muscle contraction and relaxation [129]. Exposure to PM, specifically through the production of peroxides, increases cytosolic calcium levels while depleting intracellular calcium stores, such as those within the sarcoplasmic reticulum [130]. This imbalance may impair cardiac contractility and promote hypertrophy. In murine models, exposure to PM2.5 for over four weeks resulted in histopathology, apoptosis, and mitochondrial dysfunction in the heart [131]. Additionally, in human AC16 cardiomyocytes, exposure to PM2.5, along with mitochondrial damage and intracellular calcium overload, induces cytotoxicity and hypertrophy. Furthermore, exposure to PM2.5 triggered the opening of the mitochondrial permeability transition pore, reduced the mitochondrial membrane potential, decreased the mitochondrial respiratory metabolic capacity, and decreased ATP production [131]. Another study showed that an intratracheal injection of PM2.5 induced heart damage by inducing an inflammatory response, oxidative stress, and intracellular free Ca^2+^ overload [128]. Cardiomyocytes isolated from mice exposed to PM2.5 showed increased concentrations of intracellular free Ca^2+^ and ROS, whereas treatment with N-acetylcysteine, an antioxidant reagent, reversed the increased Ca^2+^ and ROS levels, suggesting that oxidative stress contributes to increased calcium accumulation and that antioxidants may help suppress this calcium buildup [128].

### 4.2. PM Attributable Hypertension

Hypertension, characterized by persistently high blood pressure in the blood vessels, affects an estimated 1.28 billion adults aged 30–79 years worldwide [132]. It is a well-established risk factor for CVDs such as stroke, heart failure, and MI. PM-induced ROS plays an important role in the pathophysiology of hypertension. NOXs, including NOX1, NOX2, NOX4, and NOX5, are well known to generate ROS in the cardiovascular system, which amplifies signaling pathways associated with endothelial dysfunction, vascular damage, and vascular remodeling [133]. Conversely, reduced levels of NO and antioxidant enzymes can also be observed. Furthermore, antioxidants such as vitamins, NOX inhibitors, and free radical mimetics decrease oxidative stress and prevent hypertension.

A meta-analysis of 41 studies showed that long-term exposure to PM1, PM2.5, and PM10 increased the OR for hypertension by 1.27 (95% CI 1.06 to 1.52), 1.15 (95% CI 1.10 to 1.20), and 1.11 (95% CI 1.07 to 1.16), respectively [134]. A recent study on global aging and adult health conducted in six countries showed that every interquartile range (IQR) increase in annual average PM2.5 exposure (16.80 µg/m^3^) raised the HR for hypertension by 1.17 (95% CI 1.10 to 1.24). Additionally, for each IQR increase, systolic blood pressure (SBP) rose by 2.54 mmHg (95% CI 1.99 to 3.10) and diastolic blood pressure (DBP) increased by 1.36 mmHg (95% CI 1.04 to 1.68) [135]. In addition to these findings, a study conducted in urban India demonstrated a significant relationship between PM2.5 exposure and blood pressure elevations. This study evaluated both monthly and annual exposures to PM2.5. For an IQR increase in monthly PM2.5 exposure, there was a significant rise of 1.77 mm Hg (95% CI 0.97 to 2.56) in SBP and 1.17 mm Hg (95% CI 0.65 to 1.70) in DBP [136]. For annual exposures, we observed a significant increase of 3.33 mm Hg (95% CI 1.12 to 5.52) in SBP, whereas the effects on DBP, although positive, were insignificant. Additionally, the risk of developing hypertension increased by 1.53-, 1.59-, and 1.16-fold for exposure durations of 1, 1.5, and 2 years, respectively, based on the IQR differences. An IQR increase (80.25 μg/m^3^) in daily PM2.5 exposure on the day of central SBP measurement (lag 0 days) was associated with a 2.54 mm Hg (95% CI 0.92 to 4.16) elevation in central SBP in a high ambient PM2.5 concentration (100 μg/cm^3^) [137].

PM-induced hypertension occurs through several mechanisms, including inflammation and oxidative stress, autonomic nervous system dysfunction, endothelial dysfunction, vascular remodeling, and arterial stiffness, all of which contribute to vasoconstriction. In vivo experiments demonstrated that PM2.5 inhalation significantly affected blood pressure regulation. Although short-term (6 h/day for 10 weeks) exposure to PM2.5 did not alter the mean arterial pressure, distinct responses were observed during angiotensin II infusion, where the PM2.5-exposed group exhibited elevated mean arterial pressure [138]. Moreover, increased levels of superoxide (O_2_^−^) were detected in the arterial tissue; this elevation was suppressed by the NOX inhibitor apocynin and NOS inhibitor N-omega-nitro-L-arginine methyl ester [138]. These findings suggest that the rise in mean arterial pressure is closely associated with oxidative stress, potentially through the coupling of O_2_·^−^ with nitric oxide, thereby reducing nitric oxide bioavailability to NOXs. This interaction may represent a crucial mechanism underlying vascular dysfunction induced by PM2.5 exposure. Furthermore, the observed increases in SBP and DBP (12 h/day for 8 weeks of PM exposure) and smooth muscle actin dysfunction were mitigated in TLR3 knockout mice, indicating that TLR3 activation is a key mediator of the hypertensive response to PM2.5 [139]. In addition, TLR3 knockout mice showed decreased inflammation and oxidative stress in response to PM2.5.

### 4.3. PM-Attributable Atherosclerotic Cardiovascular Disease (ASCVD)

ASCVD refers to a range of conditions caused by plaque buildup in the arterial walls, including acute coronary syndromes, MI, stable or unstable angina, stroke, transient ischemic attacks, and peripheral arterial disease [140]. Numerous studies revealed a correlation between PM2.5 exposure and ASCVD mortality. Coronary artery calcium levels, carotid intima-media thickness, and plaques are used as surrogates for ASCVD [141]. A meta-analysis of eight studies (2007–2013) showed that for every 10 μg/m^3^ increase in PM2.5 exposure, carotid intima-media thickness increased by 22.52 μm, although this difference was not significant [142]. In a study conducted on 364 South Korean residents using coronary computed tomographic angiography, every 1 μg/m^3^ increase in PM2.5 was related to the development of high-risk plaque (HR 1.62, 95% CI 1.22 to 2.15). Moreover, higher exposure increased the fibrofatty or necrotic core component in newly developed plaques (HR 1.41, 95% CI 1.23 to 1.61) and contributed to total plaque volume progression in pre-existing plaques (HR 1.14, 95% CI 1.05 to 1.23) [143]. Another study of 3127 participants in Korea compared coronary artery calcium progression across the lowest, middle, and highest tertiles of PM2.5, with rates of 28.5%, 44.1%, and 57.9%, respectively, and indicated an increase in coronary artery calcium progression with higher PM2.5 [144]. The study also showed that for every 100 μg/m^3^ increase in PM2.5, the unadjusted OR was 1.08 (95% CI 1.07 to 1.09), and the adjusted odds ratio increased with cumulative exposure.

#### 4.3.1. PM-Attributable MI

MI, commonly known as a heart attack, is a severe coronary artery disease caused by the narrowing or blockage of the coronary arteries because of atherosclerotic plaque buildup [145]. MI occurs when blood flow to the heart muscle is significantly reduced or completely obstructed, leading to an imbalance between the coronary blood supply and myocardial oxygen demand, ultimately resulting in myocardial necrosis [146]. This condition is a major public health concern, particularly among the older population [147]. A systematic review and meta-analysis, which included 22 studies and 29,826,717 participants, revealed that the global prevalence of MI is 3.8% among individuals under the age of 60 years and 9.5% in those aged ≥60 years [147]. Various cohort studies and meta-analyses have explored the correlation between PM exposure and MI incidence. A meta-analysis of studies retrieved in October 2021 involving over 70 million participants demonstrated a significant association between long-term exposure to respirable PM and the development of MI, with an HR of 1.01 [148]. A study performed in Poland highlighted a significant association between air pollution and increased risks of both non-ST-elevation MI and ST-elevation MI. Notably, the risk is heightened in specific vulnerable groups including younger individuals, women, residents of rural areas, and those with lower income levels [149]. Another study was conducted using inpatient discharge data from four provinces in China (Shanxi, Sichuan, Guangxi, and Guangdong) from 2014 to 2019. The fatality rates of acute MI were examined based on the PM size. For short-term exposure (7-day average), the ORs per 10 µg/m^3^ were 1.052 (95% CI 1.03 to 1.07) for PM1, 1.026 (95% CI 1.01 to 1.03) for PM2.5, and 1.016 (95% CI 1.00 to 1.02) for PM10. For long-term exposure (annual average), the ORs were 1.303 (95% CI 1.25 to 1.35) for PM1, 1.209 (95% CI 1.17 to 1.24) for PM2.5, and 1.157 (95% CI 1.13 to 1.18) for PM10 [150]. These findings suggest that controlling PM levels is crucial for reducing the incidence and mortality rate of MI.

One mechanism through which PM exposure increases the risk of MI is the excessive production of ROS. Although moderate ROS production exerts a protective effect against myocardial ischemia, excessive ROS generation can lead to myocardial damage. Animal studies showed that PM exposure results in oxidative stress, reduced ATP levels, and mitochondrial dysfunction, which exacerbate myocardial damage, particularly in PM + MI models [151]. Particularly, the levels of antioxidants, including superoxide dismutase, catalase, and glutathione, were measured to assess oxidative stress, revealing a significant decrease in antioxidant levels [151]. In a left anterior descending coronary artery ligation-induced MI model treated with PM, there was an increase in the infarct and myocyte size, along with a reduction in myofilaments. Additionally, myocardial apoptosis was elevated, which was accompanied by the activation of NFκB [152].

#### 4.3.2. PM-Attributable Stroke

Stroke is one of the leading causes of death worldwide and is a medical emergency that can result in severe disability or death if not treated promptly [153]. Stroke occurs when blood flow to the brain is blocked or when a blood vessel in the brain bursts, leading to damage to the brain tissue [154]. Stroke is classified into two main types, namely ischemic stroke, which occurs when blood flow to the brain is obstructed (accounting for approximately 85% of all strokes), and hemorrhagic stroke, which occurs when a blood vessel in the brain ruptures (accounting for approximately 15% of strokes) [155]. Both types of strokes can cause lasting neurological deficits depending on the location of the brain damage. Stroke, particularly ischemic stroke, is caused by excitotoxicity, cell death, neuroinflammation, and oxidative stress [156]. Oxidative stress is induced by several pathways, including HIF1α, Nrf2, and CK2, leading to neuronal cell death and neuroinflammation [156]. Therefore, antioxidants have potential effects on stroke therapy. Indeed, several antioxidants could relieve stroke in preclinical studies.

Recent studies focused on the impacts of air pollution, particularly PM2.5 and PM10, on stroke (Table 2). Approximately 21% of all stroke-related deaths are attributable to air pollution [157]. Globally, PM2.5-related stroke mortality and DALYs amount to 1.14 million (95% UI 0.94 to 1.33) and 28.74 million (95% UI 23.37 to 33.44), respectively [158]. The study of the incidence of stroke differs depending on short-term and long-term exposure [159]. In the case of short-term exposure, an increase in PM10 by an IQR of 14.46 μg/m^3^ was associated with an OR of 1.26 (95% CI 1.03 to 1.55) for ischemic stroke [160]. Another meta-analysis of 94 studies conducted across 28 countries showed that short-term exposure to PM was positively correlated with stroke incidence and mortality. For every 10 μg/m^3^ increase in PM2.5, the relative risk (RR) was 1.011 (95% CI 1.011 to 1.012). In contrast, an increase in PM10 (RR 1.003, 95% CI 1.002 to 1.004) was associated with a lower RR, indicating that PM2.5 is more strongly associated with stroke hospitalization or stroke mortality [161]. The inconsistency in study results may be due to variations in hourly or daily concentrations during short-term exposure or differences related to geographical location [159]. In the case of long-term exposure, a meta-analysis of 16 cohort studies found that for every 5 µg/m^3^ increase in PM2.5, the incidence of stroke increased with an HR of 1.11 (95% CI 1.05 to 1.17), whereas stroke mortality also showed a similar HR of 1.11 (95% CI 1.05 to 1.17) [162]. Another systematic and meta-analysis showed that long-term PM2.5 and PM10 have different effects on stroke; PM2.5 is associated with total stroke (RR 1.09, 95% CI 1.04 to 1.14) and ischemic stroke (RR 1.15, 95% CI 1.06 to 1.24) but not with hemorrhagic stroke [163]. Additionally, PM10 has been suggested to have no association with ischemic or hemorrhagic stroke. In addition, a study conducted in Denmark demonstrated that a 3.9 μg/m^3^ IQR increase in the annual average of PM2.5 led to a 12% increase in the incidence of ischemic stroke [164].

In vivo studies showed that PM induces stroke via neuroinflammation. When atmospheric PM, CRM28, was administered intranasally, microglial activation (but not astrocyte activation) was observed in the olfactory bulb and cerebral cortex of mice [165]. Primary cell culture studies also confirmed that PM2.5 induces inflammation in microglia. Furthermore, PM2.5 worsens stroke prognosis by reducing retention time, microglial activation, and ischemic edema in photothrombotic-induced ischemic stroke models. In a middle cerebral artery occlusion model, PM2.5 significantly increased the infarct volume, inhibited autophagy, and reduced tight junction integrity [139]. After chamber aerosolization exposure to nanosized PM, mice subjected to middle cerebral artery occlusion/reperfusion exhibited increased infarct volumes and reduced neurological deficit scores [166]. Additionally, an increase in the density of 8-OHdG in the core of the occlusion/reperfusion area indicates heightened oxidative stress.

**Table 2:** Cohort studies of the relationship between PM2.5 and stroke.

Model/Population	Individuals	Hazard Ratio (95% CI)	Annual PM Conc. (µg/m^3^)	
Hong Kong Chinese cohort (follow-up: 9.4 years, 1998~2001 to 2010)	66,820 (mean age: 72)	Every 10 µg/m^3^ increment in PM2.5: Total stroke: 1.14 (1.02 to 1.27) Ischemic stroke: 1.21 (1.04 to 1.41) Hemorrhagic stroke: 0.90 (0.70 to 1.17), non-significant	35.8 ± 2.4	[167]
Yinzhou District, Ningbo, Zhejiang Province, China (follow-up: 4.08 years, 2015 to 2018)	27,375 (mean age: 63.09, 9.35)	Every 5 µg/m^3^ increment in PM2.5: Ischemic stroke: 1.26 (1.11 to 1.43)		[168]
Northern China (follow-up: 9.8 years, 2000~2009)	38,140 (mean age: 43.96 ± 13.68)	Every 10 µg/m^3^ increment in PM2.5: Total stroke: 1.31 (95% C: 1.04 to 1.65)	66.3	[169]
China-PAR project (follow-up: 12.06 years, 1998~2008 to 2007~2020)	40,827 (mean age: 51.83 ± 10.56)	Every 10 µg/m^3^ increment in PM2.5: Total stroke: 1.24 (1.21, 1.27) Ischemic stroke: 1.32 (1.28, 1.36) Hemorrhagic stroke: 1.12 (1.06, 1.19)	62.20	[170]
Danish Nurse Cohort (follow-up: 19.45 years, 1993 or 1999 to 2014)	23,423 (mean age: 52.6 ± 7.7)	Every IQR (3.9 µg/m^3^) increment in PM2.5: Overall: 1.12 (1.01 to 1.25) Ischemic: 1.13 (1.01 to 1.26) Hemorrhagic stroke: 1.07 (0.80 to 1.44)	19.7 ± 3.6	[164].
Taiwan (follow-up: 6.0 years, 2010~2015)	1,362,284 (mean age: 44.0 ± 15.2)	Every IQR increment in PM2.5 (IQR: 9.6, 12.1, 8.7, 8.3, 7.9, and 6.1 µg/m^3^ for 2010, 2011, 2012, 2013, 2014, and 2015): Total stroke: 1.03 (1.00 to 1.06) Ischemic stroke: 1.06 (1.02 to 1.09) Hemorrhagic stroke: 0.95 (0.89 to 1.10), non-significant	30.4 ± 7.2 in 2010 to 21.1 ± 4.5 in 2015	[171]
Canadian Census Health and Environment Cohort (CanCHEC) (follow-up: 10 years, 2006 to 2016)	Approximately 2.7 million	Every IQR (3.27 µg/m^3^) increment in PM2.5: Total stroke: 1.078 (1.052 to 1.105)	6.77 (range: 0.75–20.00)	[172]
Women’s Health Initiative (WHI) (follow-up: 15 years, 1993 to 1998~2010)	155,410 women (mean age: 63.2, 7.2)	Every IQR (3.5 µg/m^3^) increment in PM2.5: Total stroke: 1.15 (1.13 to 1.18) Ischemic stroke: 1.15 (1.12 to 1.18) Hemorrhagic stroke: 1.13 (1.06 to 1.19)	14.2 ± 2.8	[173]

## 5. PM-Associated Skin Disease

**Figure 5:**
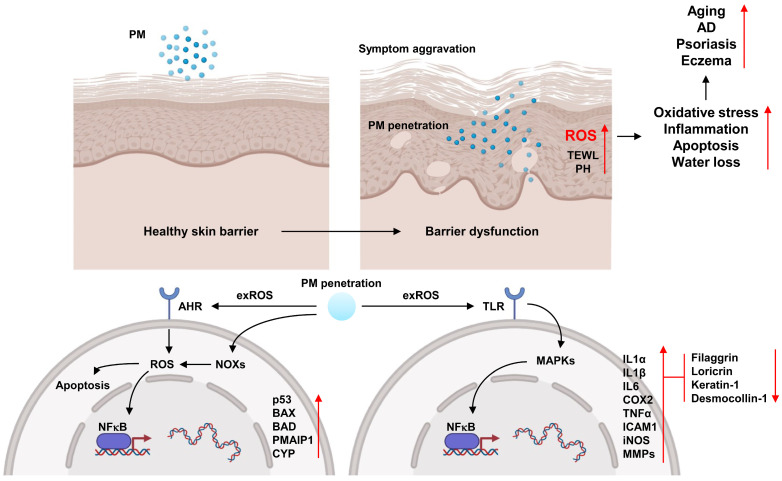
Pathogenesis of inflammatory skin disease and aging by PM exposure and associated ROS signaling pathway. Atopic dermatitis: AD; aryl hydrocarbon receptor: AhR; BCL2-associated agonist of cell death: BAD; BCL2-associated X protein: BAX; cyclooxygenase 2: COX2; cytochrome P450: CYP; exogenous ROS: exROS; intercellular adhesion molecule 1: ICAM1; interleukin: IL; inducible nitric oxide synthase: iNOS; mitogen-activated protein kinase: MAPK; matrix metalloproteinase: MMP; NADPH oxidase: NOX; nuclear factor kappa B: NFκB; occludin: OLCN; particulate matter: PM; phorbol-12-myristate-13-acetate-induced protein 1: PMAIP1; reactive oxygen species: ROS; transepidermal water loss: TEWL; Toll-like receptor: TLR; tumor necrosis factor: TNFα. Created with BioRender.com. accessed on 20 September 2024.

The skin tissue provides a defense mechanism against environmental factors such as chemicals, pathogens, air pollutants, and ultraviolet radiation and retains moisture to prevent internal moisture loss [174]. Keratinocytes, which constitute over 90% of the epidermis, interact with numerous antigens and when exposed to stressful environments, generate a variety of cytokines and chemokines, thereby participating in inflammatory and immune responses [175]. The protective function of keratinocytes against the invasion of foreign substances may be dysregulated, resulting in excessive production and damage of inflammatory mediators, which can cause skin aging and inflammation [176,177]. Upon penetrating the human body, PM generates excessive ROS, leading to oxidative stress, which induces DNA damage in skin cells, cellular apoptosis, moisture loss, skin aging, and inflammation [178] (Figure 5).

### 5.1. PM-Induced Oxidative Stress in Inflammatory Skin Disease

Globally, 25% of the population suffers from inflammatory skin diseases, including atopic dermatitis, psoriasis, eczema, and pruritus, which are typically caused and exacerbated by disruption of the skin barrier and immunological imbalances [179,180]. Although inflammatory skin diseases are more affected by genetic variants, a recent study showed that exposure to airborne pollutants, particularly PM2.5, contributes more significantly to elderly atopic dermatitis than genetic mutations [181]. A recent prospective study of the association between infantile atopic dermatitis and exposure to PM10 during early pregnancy revealed that a 10 µg/m^3^ increase in PM10 exposure during the first trimester of pregnancy was associated with a 1.21-fold (95% CI 1.02 to 1.45) increase in atopic dermatitis risk and exposure throughout the pregnancy was associated with a 1.09-fold (95% CI 0.79 to 1.51) increase in risk [182]. Furthermore, a longitudinal study assessing the clinical effects of outdoor air pollution on atopic dermatitis symptoms found that a 1 μg/m^3^ increase in PM10 was correlated with a 0.44% (95% CI 0.12 to 0.77) increase in atopic dermatitis symptoms on the following day, whereas a 1 μg/m^3^ increase in PM2.5 was associated with a 0.67% (95% CI 0.03 to 1.38) increase in atopic dermatitis symptoms the next day [183]. Kim et al. showed that 34 of 425 children under the age of 5 years diagnosed with atopic dermatitis (8%) underwent indoor air purification, which reduced the average PM10 concentration from 182.7 to 73.4 μg/m^3^ for 7 months [184]. Accordingly, this intervention resulted in a decrease in the average Eczema Area and Severity Index score from 2.37 to 1.19, and the initial prevalence of atopic dermatitis decreased from 8% to 7.06%, indicating an improvement in atopic dermatitis [184]. A recent study analyzed the association between childhood eczema and PM exposure during pregnancy and the first year of life in 3167 children [185]. This study revealed that during pregnancy, the estimated ORs for eczema associated with PM2.5 exposure at the third and fourth quartiles (73.28 to 74.63 µg/m^3^) were 1.37 (95% CI 1.08 to 1.75) and 1.45 (95% CI 1.12 to 1.86). For PM10 exposure at the third and fourth quartiles (112.82 to 124.87 µg/m^3^), the ORs were 1.29 (95% CI 1.02 to 1.63) and 1.46 (95% CI 1.11 to 1.92), respectively [185]. These results demonstrate that high levels of PM exposure during pregnancy are positively associated with an increased risk of eczema during the first year of life.

Psoriasis is a chronic relapsing condition characterized histologically by epidermal hyperproliferation and skin inflammation. The pathogenesis of psoriasis is driven by Th17-mediated immune responses, with IL17 and inflammatory infiltrates playing a critical role in the initiation and exacerbation of psoriasis [186,187]. Lan et al. revealed that every 10 µg/m^3^ increase in PM2.5 and PM10 exposure was associated with an increase in outpatient visit rates by 0.32% (95% CI 0.01 to 0.63) and 0.26% each (95% CI 0.05 to 0.48) in a time-series analysis of 13,536 outpatients with psoriasis [188]. Furthermore, a retrospective analysis using case-crossover and cross-sectional designs for patients with chronic plaque psoriasis revealed that an increase in the Psoriasis Area and Severity Index was associated with short-term exposure to PM during the 60 days before assessment [189]. Exposure to PM10 levels above 20 μg/m^3^ and PM2.5 levels above 15 μg/m^3^ for 60 days resulted in an increase in the Psoriasis Area and Severity Index by 1.55% (95% CI 1.21 to 1.99) and 1.25% (95% CI 1.00 to 1.57), respectively [189].

The pathogenic mechanisms of air pollutants in inflammatory skin diseases primarily involve exogenous ROS, oxidative stress-mediated inflammatory responses, and skin barrier damage [178]. PAHs, the major components of PM, bind to the aryl hydrocarbon receptor in the cytoplasm of keratinocytes, form a heterodimer complex, and translocate to the nucleus, thereby inducing the production of CYP [178]. CYP-mediated metabolism leads to the generation of quinones and ROS, which can induce the production of inflammatory cytokines such as TNFα, IL6, IL8, IL17, and IL23 [69]. Moreover, PM induces the generation of ROS, which activate MAPK signaling pathways, including ERK1/2, c-Jun N-terminal kinases, and p38 MAPK, and subsequently activate various transcription factors such as NFκB and AP-1, thereby leading to an increased production of cytokines associated with inflammatory skin diseases, such as IL1α, IL6, IL8, and TNFα, as well as matrix metalloproteinases (MMPs) including MMP1, MMP2, MMP3, and MMP9, along with exacerbating skin inflammation and damage [174,178]. Recent studies showed that exposure to PM2.5 increases ROS production and inhibits glutathione synthesis, leading to cellular damage and oxidative stress [190], and upregulates the expression of apoptosis-related markers, including Bcl-2-associated X protein, caspase-3 and caspase-9 through mitochondrial membrane depolarization and ERK activation in keratinocytes [191]. Piao et al. showed that exposure to PM2.5 induces oxidative stress in keratinocytes through the generation of ROS, leading to DNA damage, lipid peroxidation, protein carbonylation, and mitochondrial membrane depolarization, thereby promoting cellular damage and apoptosis [192]. Furthermore, PM exposure promotes the production of ROS and the generation of inflammatory cytokines such as IL1β, IL6, and COX2 via the MAPK pathway in keratinocytes and mice, leading to hyperkeratosis, mast cell infiltration, and a reduction in skin barrier function [193]. Ryu et al. revealed that PM2.5 exposure in keratinocytes enhances NFκB transcription and activation through the TLR5 and MyD88 signaling pathways, promoting IL6 production via DNA methylation and histone methylation and inducing excessive ROS generation through NOX4 activation, leading to monocyte accumulation and inflammation development in skin tissue [194]. Additionally, Lee et al. demonstrated that PM exposure promotes ROS generation via the NCF1, gp91phox, and aryl hydrocarbon receptor signaling pathways; therefore, ROS-mediated activation of the ERK1/2, p38/NFκB, and c-Jun N-terminal kinases/AP-1 signaling cascades increases COX2 and prostaglandin E2 production while downregulating filaggrin, leading to impaired skin barrier function in both keratinocytes and mice [195].

### 5.2. PM-Induced Oxidative Stress in Skin Aging

Skin aging caused by environmental factors such as PM and sun exposure is referred to as extrinsic skin aging and is characterized by deep coarse wrinkles, pigment irregularities, and solar elastosis [196]. Vierkotter et al. conducted a cohort study to analyze intrinsic and extrinsic skin aging scores in 400 Caucasian women aged 70–80 years. The results revealed that exposure to PM was directly associated with skin aging, specifically an increase in nasolabial folds and irregular pigmentation spots on the face [197]. A recent study showed that PM exposure in a reconstructed human epidermis increases inflammatory responses, including cytokines IL1α and IL8, as well as cytotoxicity, oxidative stress, and expression of the related genes MT1G and MT1E; PM also induces desquamation and apoptosis in the skin tissue, ultimately accelerating skin aging [198]. Additionally, ultraviolet radiation and PM-containing PAHs upregulate the expression of the pigmentation-related gene proopiomelanocortin through an aryl hydrocarbon receptor-dependent mechanism in keratinocytes and stimulate melanin synthesis in melanocytes, thereby leading to lentigo, which is evidence of skin aging induced by PM exposure [199]. Recently, Lee et al. showed that PM10 exposure promotes ROS generation in HaCaT cells via activation of the ERK and p38 MAPK pathways, leading to the upregulation of apoptosis-related genes such as Bcl-2 and Bcl-2-associated X protein, resulting in skin cell damage and accelerating the aging process [200].

## 6. PM-Associated Neurodegenerative and Neurodevelopmental Impairment

PM is associated with cardiovascular and respiratory conditions. It is also closely related to neurological disorders, including dementia, cognitive impairment, stroke, Parkinson’s disease (PD), and mental disorders such as depression. A longitudinal cohort study across the United States confirmed that the risk of hospital visits related to PD or Alzheimer’s disease and related dementia increases with increasing PM2.5 [201]. Notably, this increase in risk was observed even at concentrations lower than the current annual standard of 12 μg/m^3^, highlighting the urgent need to reduce PM exposure to prevent central nervous system (CNS) impairment. According to a study by the Monongahela-Youghiogheny Healthy Aging Team, for every 1 μg/m^3^ increase in annual average PM2.5 concentrations, the HR for incident dementia increased by 67% (HR 1.669, 95% CI 1.29 to 2.13) and for incident mild cognitive impairment by 75% (HR 1.746, 95% CI 1.51 to 2.03) [202]. Furthermore, with a 1 μg/m^3^ increase in the 5-year average PM2.5 concentrations, the incidence of dementia more than doubled (HR 2.082, 95% CI 1.52 to 3.01), whereas the incidence of mild cognitive impairment more than tripled (HR 3.419, 95% CI 2.80 to 4.16). Another meta-analysis demonstrated that PM2.5 was associated with increased risks for dementia (HR 1.14, 95% CI 1.14 to 1.47), Alzheimer’s disease (HR 1.65, 95% CI 1.37 to 1.94), and PD (HR 1.17, 95% CI 1.00 to 1.33) [203]. These studies suggested that PM is closely associated with CNS impairment.

### 6.1. Mechanisms of Neurodegenerative and Neurodevelopmental Impairment Induced by PM

**Figure 6:**
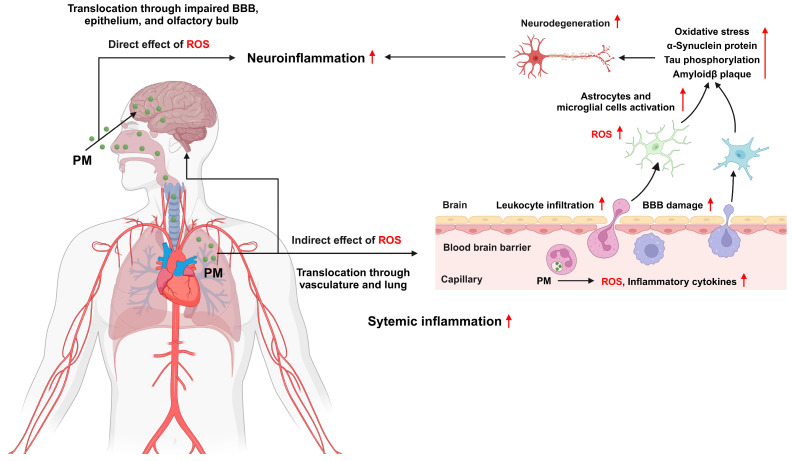
Effects and major pathways of ROS-mediated pathophysiology on neurodegenerative diseases, mental disorders, and impairment of brain development following PM exposure. Blood–brain barrier: BBB; particulate matter: PM; reactive oxygen species: ROS. Created with BioRender.com. accessed on 20 September 2024.

PM can affect the brain through both direct and indirect pathways [204]. After inhalation through the nose, PM enters the brain directly by traveling along the olfactory nerve. PM2.5 can also reach the brain by crossing the blood–brain barrier (BBB) through the bloodstream. Animal studies and autopsy reports confirmed that PM can accumulate in brain tissues, induce neuroinflammation, and damage the CNS [205,206]. Indirect PM inhalation into the lungs can trigger systemic inflammatory responses that ultimately affect the brain through the circulatory system. Substantial evidence links PM exposure to neurological disorders. The mechanisms by which PM causes neurological impairments include oxidative stress, neuroinflammation, and BBB disruption. PM exposure can cause behavioral abnormalities in experimental animals, which occur alongside oxidative stress and neuroinflammation [207,208]. For example, rats exposed to PM in a chamber for 60 min daily for two weeks exhibited behavioral abnormalities, such as memory impairment, anxiety-like and depression-like behaviors, and reduced locomotor and exploratory activities [207]. This effect was accompanied by increased BBB permeability and edema and reduced antioxidant activity in the cerebral cortex and hippocampus, as evidenced by decreased superoxide dismutase and glutathione peroxidase levels and increased MDA levels, indicating oxidative stress in the brain. Another study confirmed similar findings in which rats injected with PM via the intratracheal route exhibited behavioral changes and increased oxidative stress in the brain [208]. In adult mice, nanoscale PM exposure for three weeks led to oxidative stress and inflammatory responses in the olfactory neuroepithelium and olfactory bulb [209]. Short-term exposure to PM2.5 causes cognitive impairment and activates astrocytes and microglia [150]. Using an in vitro BBB model, Kang et al. showed that PM2.5 can permeate the BBB, leading to astrogliosis, which in turn causes neuronal loss and microglial infiltration [210]. The infiltrated microglia are then polarized into the M1 phenotype by IL1β and IFNγ. M1 microglia secrete proinflammatory mediators and NO, which contribute to neuronal damage. Another study showed that PM impairs long-term potentiation in the hippocampal dentate gyrus [211]. PM exposure induces oxidative stress and an inflammatory response, leading to brain edema and neuroinflammation, which are associated with neurodegenerative and neurodevelopmental impairment (Figure 6).

### 6.2. PM-Attributable Dementia and Cognitive Impairment

Dementia is characterized by cognitive impairments affecting memory, thinking, and behavior, making daily activities difficult [212]. Treatment of dementia is associated with high social and economic costs. As the global population ages, the number of people with dementia is rapidly increasing. In 2021, Alzheimer’s disease and other forms of dementia were ranked as the seventh leading causes of death, killing 1.8 million people [213]. Women are disproportionately affected, with 68% of deaths from Alzheimer’s disease and other forms of dementia occurring in women globally [214]. According to the 2019 Global Burden of Disease Study, the number of people with dementia was estimated to be 57.4 million in 2019 and is projected to increase to 152.8 million by 2050 [212]. Numerous studies indicated that exposure to PM2.5 plays an important role in the development of dementia and cognitive impairment. Several meta-analyses focused on the link between PM2.5 exposure and dementia risk. A systematic review and meta-analysis showed that PM2.5 exposure contributes to age-related cognitive impairment and dementia [215]. The pooled risk estimate indicated that a 5 μg/m^3^ increase in PM2.5 was associated with an HR of 1.26 (95% CI 1.18 to 1.35) for dementia, whereas the OR for cognitive impairment was 1.17 (95% CI 1.07 to 1.27). Another systematic review and meta-analysis of long-term PM2.5 and dementia showed that prolonged exposure to PM2.5 increases the risk of Alzheimer’s disease and vascular dementia [216]. Specifically, a 10 μg/m^3^ increase in PM2.5 levels was associated with an HR of 1.4 for dementia and 2.0 for vascular dementia. A systematic review and meta-analysis of 20 studies showed that the risk of dementia increased by 3% per 1 μg/m^3^ increase in PM2.5 (HR 1.03, 95% CI 0.99 to 1.13) [217]. Table 3 provides an overview of several cohort studies investigating the association between PM2.5 exposure and dementia. Although the exact mechanisms by which PM2.5 contributes to dementia are not fully understood, current research suggests that neuroinflammation, oxidative stress, and amyloid beta (Aβ) deposition are the primary pathways.

Alzheimer’s disease, the main cause of dementia, is characterized by the presence of extracellular Aβ 1–42 plaques, neurofibrillary tangles, and intraneuronal tau aggregations. PM2.5 is also considered an important risk factor for Aβ pathology and the phosphorylation of Tau [218,219,220]. For instance, a study of 18,178 individuals revealed that those living in areas with high PM2.5 levels had significantly higher Aβ plaque deposition in the brain, as measured by amyloid positron emission tomography scans [219]. In addition, intracellular Aβ 1–42 and phospho-Tau levels were increased in mice exposed to PM compared to those exposed to filtered air [220]. Amyloid precursor protein plays a crucial role in the production of Aβ. Previous studies showed that exposure to PM accelerates the conversion of amyloid precursor protein into Aβ. An analysis of an amyloid precursor protein/PS1 transgenic mouse model showed that PM2.5 exposure significantly increased Aβ plaque deposition in the hippocampus compared to filtered air, and this increase was accompanied by elevated levels of astrocytosis and microgliosis, both of which are associated with neuroinflammation [218]. These findings suggest that PM2.5, which accelerates amyloid pathology and exacerbates neuroinflammatory responses, contributes to the pathophysiology of Alzheimer’s disease. Intranasal administration of diesel exhaust particles (DEPs) impairs learning and memory functions; increases ROS levels; and leads to microglial activation, the inhibition of mitochondrial gene expression, and NLRP3 activation [217]. ROS plays a crucial role in NLRP3 activation. Interestingly, in NLRP3 knockout mice, despite DEP treatment, learning and memory functions were not impaired [217]. This result indicated that the microglia-mediated NLRP3 inflammasome plays a key role in PM-induced cognitive impairment.

**Table 3:** Cohort studies of the relationship between PM2.5 and dementia.

Model/Population	Individuals	Hazard Ratio (95% CI)	Annual PM Conc. (µg/m^3^)	
Hong Kong Chinese cohort (follow-up: 10.4 years, 1998~2001 to 2011)	66,820 (mean age: 72)	Every IQR (3.8 µg/m^3^) increment in PM2.5: All-cause of dementia: 1.06 (1.00 to 1.13) Alzheimer’s disease: 1.03 (0.94 to 1.12) Vascular dementia: 1.09 (0.98 to 1.22)	35.2 ± 3.2	[221]
Rome (follow-up: 10.6, 2001 to 2013)	350,844 (mean age: 74.5 ± 6.8)	Every 5 µg/m^3^ increment in PM2.5: Dementia: 0.99 (0.96 to 1.02) Vascular dementia: 1.07 (1.01 to 1.12) Alzheimer’s disease: 0.91 (0.85 to 0.97)	19.7 ± 2.0	[222]
Northeastern U.S. (1999 to 2010)	9.8 million (mean age: 75.6 ± 7.6)	Every 1 µg/m^3^ increment in PM2.5: Dementia: 1.08 (1.05 to 1.11) Alzheimer’s disease: 1.15 (1.11 to 1.19) Every 5 µg/m^3^ increment in PM2.5: Dementia: 1.46 (1.29 to 1.66) Alzheimer’s disease: 2.00 (1.70 to 2.35)	12.0 ± 1.6	[223]
Environmental Predictors of Cognitive Health and Aging (EPOCH) cohort study (follow-up: 10.2 years, 1998 to 2016)	27,857 (mean age: 61 ± 10)	Every 1 µg/m^3^ increment in PM2.5: Dementia: 1.02 (1.00 to 1.04)	11.2 ± 3.7	[224]
United States (follow-up: ~years, 2000 to 2017)	18.5 million for dementia, 19.2 million for AD	Every IQR (3.68 µg/m^3^) increment in PM2.5: Dementia: 1.068 (1.063 to 1.072) Alzheimer’s disease: 1.106 (1.099 to 1.114) Every 1 µg/m^3^ increment in PM2.5: Dementia: 1.018 (1.017 to 1.019) Alzheimer’s disease: 1.028 (1.026 to 1.030)	9.58	[225]
Taiwan (follow-up: 5.18 years, 2001~2004 to 2013)	1,362,284 (mean age: 73.4 ± 10.4)	Every IQR increment in PM2.5 (IQR 9.6, 12.1, 8.7, 8.3, 7.9, and 6.1 µg/m^3^ for 2010, 2011, 2012, 2013, 2014, and 2015): Total stroke: 1.03 (1.00 to 1.06) Ischemic stroke: 1.06 (1. 02 to 1.09) Hemorrhagic stroke: 0.95 (0.89 to 1.10), non-significant.	30.4 ± 7.2 in 2010 to 21.1 ± 4.5 in 2015	[226]
French (follow-up: 10 years, 1999~2001 and followed for 12 years)	7066 (mean age: 73.4 ± 8.0)	Every 5 µg/m^3^ increment in PM2.5: All-cause dementia: 1.20 (1.08 to 1.32) Alzheimer’s disease: 1.20 (1.09 to 1.32) Vascular dementia: 1.33 (1.05 to 1.68)	21.9 ± 2.6	[227]

### 6.3. PM-Attributable Neurodevelopmental Impairment

PM can affect the fetus by crossing the placenta. An analysis of umbilical cord blood at birth revealed prenatal exposure to PM2.5, indicating that PM can penetrate the placental tissue [228]. The prenatal period is particularly sensitive to neurotoxicants and exposure during this critical period can have lifelong consequences. For instance, a study conducted in Mexico demonstrated that exposure to ambient PM2.5, especially in the first trimester during pregnancy, was associated with neurobehavioral development in children aged 4–6 years [229]. Brain-derived neurotrophic factor (BDNF) plays a crucial role in nervous system development. Exposure to PM2.5 during both the first and second trimesters was associated with reduced BDNF levels [228,230]. Specifically, PM2.5 exposure during the second trimester was negatively correlated with BDNF levels at birth [228]. A one natural log unit increase in maternal PM2.5 exposure during the second trimester was linked to a −0.2 log unit decrease in BDNF at birth (95% CI −0.36 to −0.05). In vivo studies showed that PM exposure during embryonic development induces neurobehavioral changes [231,232,233]. Pregnant mice were exposed to either concentrated ambient PM2.5 or filtered air. Offspring exposed to PM2.5 exhibited impaired learning and memory at 6 weeks of age, as demonstrated in the Morris water maze test [231]. Additionally, microglia were increased in the cerebellum of the PM2.5-exposed offspring. Furthermore, the oxidative nucleic acid modification marker 8-OHdG was elevated in microglia and Purkinje cells isolated from the cerebral tissue. The cerebellar tissue also showed increased levels of the oxidative stress marker 8-iso-prostaglandin F2α, as well as the inflammatory cytokines IL8 and TNFα [231].

### 6.4. PM-Attributable Depression

Depression is a serious mental health concern worldwide. In 2019, an estimated 280 million people worldwide, accounting for 5% of all adults, experienced depression [234]. A systematic review and meta-analysis of 72 studies published between 2001 and 2020 of adolescents aged 10–19 years found that 34% (95% CI 0.30 to 0.38) self-reported depressive symptoms [235]. Depression can arise through a variety of mechanisms, including neuroinflammation, dysregulation of the hypothalamic–pituitary–adrenal axis, imbalances in neurotransmission, impaired neurogenesis, and altered synaptic plasticity [236]. Oxidative stress plays a crucial role in influencing these pathways, potentially exacerbating the onset and progression of depression. Depression is also associated with exposure to PM. A systematic review and meta-analysis conducted using data from 39 studies up to May 2021 demonstrated a significant relationship between PM2.5 exposure and depression [237]. Another systematic review and meta-analysis showed that long-term exposure to PM2.5 is significantly associated with an increased RR of depression (1.074, 95% CI 1.02 to 1.12). In addition, short-term PM2.5 (RR 1.009, 95% CI 1.00 to 1.01) and PM10 exposure (RR 1.009, 95% CI 1.00 to 1.01) were also significantly associated with depression. For every 10 μg/m^3^ increase in short-term PM2.5 exposure, there was a 2% increase in the risk of depression and a 2% increase in the risk of suicide [238]. Long-term exposure to a 10 μg/m^3^ increase in PM2.5 was linked to an 18% increase in the risk of depression. Similarly, short-term exposure to PM10 resulted in 2% and 1% increases in the risk of depression and suicide, respectively. A study conducted in South Korea, as part of the Korean Longitudinal Study of Aging from 2016 to 2020, showed that a 10 μg/m^3^ increase in long-term PM2.5 exposure was associated with an increased risk of depression (OR 1.36, 95% CI 1.20 to 1.56), as was similar with an increase in PM10 exposure (OR 1.19, 95% CI 1.10 to 1.29) in the elderly [239].

In vivo studies demonstrated that PM2.5 exposure can induce depression. For example, PM2.5 exposure led to depressive behaviors in mice and inhibited electroencephalogram activity, as assessed in an open field test and elevated plus maze [240]. Exposure to PM2.5 for 8 weeks resulted in increased microglial activity in the brain tissue through the MAPK/CREB/BDNF pathway. An in vitro analysis using PC12 cells showed that PM2.5 induces oxidative stress [240]. Another study revealed that 12 weeks of exposure to DEPs not only induced depression- and anxiety-like behaviors but also impaired learning and memory functions [241]. In addition, neuroinflammation was observed in the hippocampi of mice exposed to DEPs. Recent studies also showed that PM2.5 interacts with dopamine receptors, disrupts dopamine signaling, and contributes to the development of mental disorders, including depression [242].

### 6.5. PM-Attributable PD

PD is a neurodegenerative disorder of the CNS, primarily characterized by motor impairments, with dopaminergic neuron loss and the abnormal accumulation of a protein called α-synuclein as the main pathological features [243]. ROS plays a significant role in PD pathology through various mechanisms [244]. Key molecules involved in the pathogenesis of PD, such as α-synuclein, PINK1, Parkin, DJ-1, and LRRK2, are regulated by ROS [245,246]. Neuroinflammation can activate NOXs, promote ROS production, and exacerbate PD [247]. Recent studies focused on the associations among air pollution, PM2.5, and PD. A meta-analysis of studies conducted up to 2021 reported that every 1 μg/m^3^ increase in PM2.5 concentration was associated with a 1% increase in the risk of developing PD (OR 1.01, 95% CI 0.99 to 1.03) [248]. Additionally, a large-scale study of 65,180 patients with PD showed that each 1 μg/m^3^ increase in PM2.5 concentration led to a 1.042 increase in the RR, which was significant (95% CI 1.03 to 1.04) [249]. These findings indicated a strong correlation between PM exposure and PD onset. In animal models used to study PD, two commonly employed experimental methods are the rotarod and adhesive tape removal tests to evaluate motor impairments in PD models. Notably, animal models exposed to PM alone exhibit abnormal motor behaviors. Moreover, when PM exposure was combined with rotenone-induced PD, these motor abnormalities worsened, indicating that PM exposure exacerbates symptoms [250]. PM-induced oxidative stress, apoptosis, and autophagy inhibition in the nigra of a mouse model showed a similar effect [250]. PM exposure has been linked to increased ROS levels, which in turn leads to the abnormal accumulation of α-synuclein, a hallmark of PD. In a study with A53T mice (a PD model), an intranasal administration of PM2.5 induced the propagation of α-synuclein inclusions [251]. After three months of exposure, α-synuclein pathology was observed in several brain regions, including the olfactory bulb, prefrontal cortex, hippocampus, amygdala, substantia nigra, and locus coeruleus. Autophagy, a cellular process responsible for clearing damaged proteins and organelles, is crucial for maintaining neuronal function. Impaired autophagy is a key pathological feature of PD. PM exposure inhibited autophagy and mitophagy by activating the mTOR pathway and inhibiting the PINK1/Parkin pathway [250]. In addition, the autophagy enhancer rapamycin can relieve the damaging effects of PM2.5 by inducing autophagy and mitophagy [250]. In studies using human glioblastoma LN-229 cells, PM exposure increased ROS production, autophagy, and apoptosis. When cells are treated with catalase (a ROS scavenger) or their mitochondrial DNA is depleted, PM-induced autophagy and apoptosis are significantly reduced [252], indicating that PM exposure induces autophagy and apoptosis via mitochondrial ROS production.

## 7. Conclusions

Over the past two decades, a wide range of in vitro animal models and epidemiological studies have been conducted to enhance our understanding of the adverse health effects associated with air pollution and ambient PM inhalation. Notably, DNA damage mediated by ROS, inflammation, and dysregulated immune responses driven by oxidative stress have emerged as potent indicators of PM biotoxicity. As highlighted in this review, PM has a variable composition and particle size because of environmental factors, such as the region of origin and meteorological conditions, which can influence the types of biological damage induced by PM exposure. Furthermore, the various mechanisms by which PM induces oxidative stress must be considered given that these processes may become further subdivided and complex within the human body depending on the exposure conditions, such as the penetration route and duration of PM exposure. Further research is needed to accurately determine the complexity of the mechanisms and related effects induced by PM-mediated oxidative stress in the human body through multi-approach analyses, including real-time and high-throughput assessments of a broad biomarker panel encompassing various related pathways.

## Figures and Tables

**Table 1:** Specifications of certified respirable materials used to describe the adverse effects of PM.

Manufacturer	SRM	Description	Features	Particle Size	Certified Measurands	Reference Measurands
National Institute of Standards and Technology	SRM 1648A	Urban particulate matter	Atmospheric particulate matter collected from the St. Louis, MO, area	d (0.5) ^a^: 5.85 μm d (0.1) ^b^: 1.35 μm d (0.9) ^c^: 30.1 μm	25 elements 21 PAHs 7 PCBs	8 elements 54 PAHs 16 nitro-PAHs 46 PCBs 34 pesticides
	SRM 1649B	Urban dust	Atmospheric particulate matter collected from the Washington, DC, area	d (0.5) ^a^: 24.3 μm d (0.1) ^b^: 7.07 μm d (0.9) ^c^: 66 μm	3 Elements 23 PAHs 13 PCBs 4 Pesticides	5 elements 38 PAHs 16 nitro-PAHs 49 PCBs 1 PBDE 34 pesticides
	SRM 1650B	Diesel particulate matter	Diesel particulate matter obtained from the Coordinating Research Council, Inc. Atlanta, GA, area	d (0.5) ^a^: 0.18 μm d (0.1) ^b^: 0.12 μm d (0.9) ^c^: 0.33 μm	26 PAHs	59 PAHs 23 nitro-PAHs 96.73% alpha quartz
	SRM 1975	Diesel particulate extract	Diesel particulate material obtained from M.E. Wright of the Donaldson Company, Inc., Minneapolis, MN, area	d (0.5) ^a^: 19.4 μm d (0.1) ^b^: 5.3 μm d (0.9) ^c^: 70 μm	8 PAHs	23 PAHs 19 nitro- PAHs
	SRM 2585	Organic contaminants in house dust	Dust obtained from vacuum cleaner bags, cleaning services, motels, and hotels in the states of North Carolina, Maryland, Ohio, New Jersey, Montana, and Wisconsin	Not provided, >90 μm	33 PAHs 30 PCBs 15 PBDEs 4 Pesticides	33 PAHs 12 PCBs 3 PBDEs 10 pesticides 5 polycyclic musk 8 PFAAs
	SRM 2786	Fine atmospheric particulate matter	Atmospheric particulate material collected in 2005 from an air intake filtration system of a major exhibition center in Prague, Czech Republic	Mean particle diameter < 4 µm	25 PAHs 2 PBDEs 8 Elements	27 PAHs 7 nitro-PAHs 5 PBDEs 13 elements 3 sugars 12 dioxins 15 furans
	SRM 2787	Fine atmospheric particulate matter	Atmospheric particulate material collected in 2005 from an air intake filtration system of a major exhibition center in Prague, Czech Republic	Mean particle diameter < 10 µm	23 PAHs 2 PBDEs 7 Elements	23 PAHs 7 nitro-PAHs 5 PBDEs 14 elements 3 sugars 12 dioxins 15 furans
	SRM 2975	Diesel particulate matter	Diesel particulate matter collected from the Minnesota, MI, area	d (0.5) ^a^: 19.4 μm d (0.1) ^b^: 5.3 μm d (0.9) ^c^: 70 μm	10 PAHs	36 PAHs 16 nitro-PAHs
National Institute for Environmental Studies	CRM 28	Urban Aerosol	Atmospheric particulate matter collected on filters in a central ventilating system in a building in Beijing city center	The diameters of 99% of particles were less than 10 μm	18 Elements	8 PAHs 16 elements

Polybrominated diphenyl ether: PBDE; polycyclic aromatic hydrocarbon: PAH; nitro-substituted PAH: nitro-PAH; polychlorinated biphenyl: PCB; standard reference material: SRM; ^a^ d (0.5), ^b^ d (0.1), and ^c^ d (0.9) show the particle size distribution parameters, indicating particle sizes below 50%, 10%, and 90% of the volume, respectively.

## Data Availability

Not applicable.
